# Pain knowledge and attitudes of final-year medical students at the University of Cape Town: A cross-sectional survey

**DOI:** 10.4102/phcfm.v12i1.2306

**Published:** 2020-07-30

**Authors:** Blessing Mashanda-Tafaune, Janieke van Nugteren, Romy Parker

**Affiliations:** 1 Department of Anesthesia and Perioperative Medicine, Faculty of Health Sciences, University of Cape Town, Cape Town, South Africa

**Keywords:** pain knowledge, attitudes, medical students, University of Cape Town, pharmacological aspects of pain management

## Abstract

**Background:**

Pain is the most common presenting complaint in patients visiting a healthcare facility. Healthcare professionals need adequate knowledge of pain to be able to manage it effectively.

**Aim:**

The aim of this study was to determine the pain knowledge and attitudes of the 2018 final-year medical students at the University of Cape Town (UCT).

**Setting:**

This study was conducted by the Department of Anaesthesia and Perioperative Medicine in the Faculty of Health Sciences, UCT, South Africa, with final-year medical students.

**Methods:**

Unruh’s Modified Pain Knowledge and Attitudes (MPKA) questionnaire was utilised to collect data in a cross-sectional survey using an Internet-based electronic format.

**Results:**

A total of 104 students out of 232 students in the class (44.8%) participated in the study. The total median score on the MPKA questionnaire was 46 (interquartile range [IQR] 44–50.5) out of 57, or 80.7% (IQR 77.2–88.6%). The participants performed worst in the section on the pharmacological management of pain with median scores of 6 (IQR 4–8) (55%) correct out of 11 questions.

**Conclusion:**

Pain knowledge, especially with regard to the pharmacological aspects of pain management, has some important deficiencies in these final-year medical students. It appears that the undergraduate curriculum and teaching thereof would benefit from a review of the pain curriculum.

## Introduction

Pain is one of the most common symptoms experienced by patients in both the inpatient and outpatient setting. However, pain knowledge amongst medical students and other health professionals is poor.^1^ The World Health Organization (WHO) has estimated that worldwide 22% of primary care patients suffer from chronic debilitating pain, with chronic pain accounting for one-fifth of physician’s visits.^2,3,4^ In South Africa (SA), 75% of patients attending an Eastern Cape rural and peri-urban health clinic visited the clinic because of pain,^5^ and in a survey of 1066 patients attending primary healthcare facilities in South-West Tshwane (SA), the prevalence of chronic pain was 41%.^6^

Acute post-surgical pain remains poorly managed.^7^ Suboptimal treatment of acute pain may increase the risk of developing chronic pain.^8^ Poorly managed postoperative pain affects both physiological and psychological function and is associated with increased morbidity, impaired function, and quality of life, delayed recovery time, prolonged use of opioids and higher hospital costs.^9^ Uncontrolled acute pain may also cause restricted breathing, leading to pulmonary complications, while immobilisation may lead to thrombi formation and increased mortality.^10^ Two national surveys in the United States reported that 80% of patients experienced severe acute pain post-surgery in 2003, and the number subsequently increased to 86% in 2013.^11,12^

Healthcare professionals need adequate knowledge of pain to be able to manage it effectively. However, pain education at medical schools is limited, variable and fragmented with pain topics being typically presented as part of general required courses.^13^ In US medical schools, many topics included in the International Association for the Study of Pain (IASP) core curriculum receive little or no coverage.^13^ In 2012, Vadivelu et al. reported that pain education had only been incorporated as part of the curriculum in 3% of schools in the United States and that medical school curricula needed to incorporate pain diagnosis and management modules.^10^ A 2017 editorial stated that in 2008, the United States identified the under-treatment of chronic pain and recommended that undergraduate medical students be exposed to pain medicine. This has not yet occurred in the majority of American medical schools.^14^

This poor knowledge of pain is not restricted to healthcare professionals training in the United States. A questionnaire completed by German physicians specialising in pain therapy revealed poor knowledge of the WHO recommendations for the treatment of cancer pain, which included the WHO analgesic ladder, with deficiencies particularly in the knowledge of pharmacological aspects.^15^ Pain management education in the United Kingdom takes up less than 1% of university teaching time for healthcare professionals.^10^ A UK survey of 11 major universities showed that the average content of pain teaching for undergraduates was 12 h.^16^ The APPEAL study on pain education curricula within undergraduate medical studies during 2012–2013 (surveying 15 European countries) showed that 55% of the medical schools taught pain within compulsory non-specific pain modules and 31% of medical schools taught pain in dedicated pain modules.^17^ Several studies in Brazil have identified that physicians have a limited understanding of pain and its assessment tools.^18^ This lack of knowledge and misconceptions regarding analgesic and opioid prescriptions leads to inadequate treatment.^18^ In Ethiopia, only 4% of medical students and paramedics scored above the cut-off point of 70% for good knowledge regarding pain management.^19^

The situation in SA appears to be similar to other countries. While no articles could be found reporting on the amount of time committed to pain education in the South African undergraduate curricula, a 2007 study at the University of Cape Town (UCT) reported on the pain knowledge and attitudes of the final-year health science students.^20^ This study showed that final-year health science students had poor knowledge of pain, with 40% of the final-year medical students scoring less than 75% on Unruh’s Modified Pain Knowledge and Attitudes Questionnaire (MPKAQ).^20^ A 2016 survey of University of Stellenbosch fifth-year medical students found that chronic pain knowledge was insufficient with respondents scoring 41%, with 74% of the respondents scoring less than 50%.^21^

Given the prevalence and negative impact of pain, having sufficient knowledge of and appropriate attitudes towards pain management are critical competencies for South African doctors. We conducted a cross-sectional survey to assess the pain knowledge and attitudes of the 2018 final-year medical students at UCT using a questionnaire-based approach.

## Methods

A cross-sectional survey of final-year medical students was conducted. To ensure a representative sample, a sample size calculation was performed using the Yamane formula:

$$n=\frac{N}{1+N{\left(e\right)}^{2}}$$ [Eqn 1]

where *N* is the study population, *e* is the constant equal to 0.05 (95% confidence) or 0.1 (90% confidence), and *n* is the sample size.^22^ There were 232 registered students in the population. Based on the Yamane formula, we aimed to recruit 147 students (95% confidence) with a minimum of 70 students (90% confidence).

All 232 registered final-year medical students were contacted via their official university email addresses and invited to participate in an online survey of pain knowledge using Unruh’s MPKAQ.^23^ This tool covers a wide base of knowledge appropriate for healthcare professionals. The Pain Knowledge and Attitudes Questionnaire has established content validity and acceptable internal consistency (Cronbach’s alpha 0.65).^24^ The questionnaire was previously adapted to make it more appropriate for a South African cultural context, and it was thus called the ‘Modified Pain Knowledge and Attitude Questionnaire’ or MPKAQ.^20^ Changes made included removing ambiguous or repeated questions and a change in the scoring from a Likert scale which was converted to a correct/incorrect response for scoring, to a simple true or false answer system. The South African adaptation of the MPKAQ was previously piloted and had comparable results to the original questionnaire.^20^

In the email sent to final-year students to recruit them to the study, detailed information about the nature of the study, its purpose, potential risks or benefits and clarifying that participation was voluntary was given. The completion of the online questionnaire implied consent to participate. The Unruh’s MPKAQ is divided into six sub-sections, which includes assessment of (1) physiological basis of pain, (2) psychological factors, (3) developmental changes of pain perception, (4) assessment and measurement of pain, (5) pharmacological management of pain and (6) cognitive or behavioural methods of pain relief.

### Data management and statistics

Given the non-parametric characteristics of the data from the MPKAQ, the data are summarised as median and interquartile range (IQR) throughout.

### Ethical consideration

This study was granted ethical approval from the UCT, Faculty of Health Sciences Human Research Ethics Committee (Ref #120/2018) and adhered to the principles of the Declaration of Helsinki throughout.

## Results

The participants in this study were final-year medical students at UCT, completing their sixth year of study. Of 232 students invited to participate, 104 students (44.8%) completed the survey. The median age of the participants was 24 years (IQR: 23–25) with 69 females (66%) taking part.

### Scores on the unruh’s modified pain knowledge and attitudes questionnaire

**FIGURE 1:**
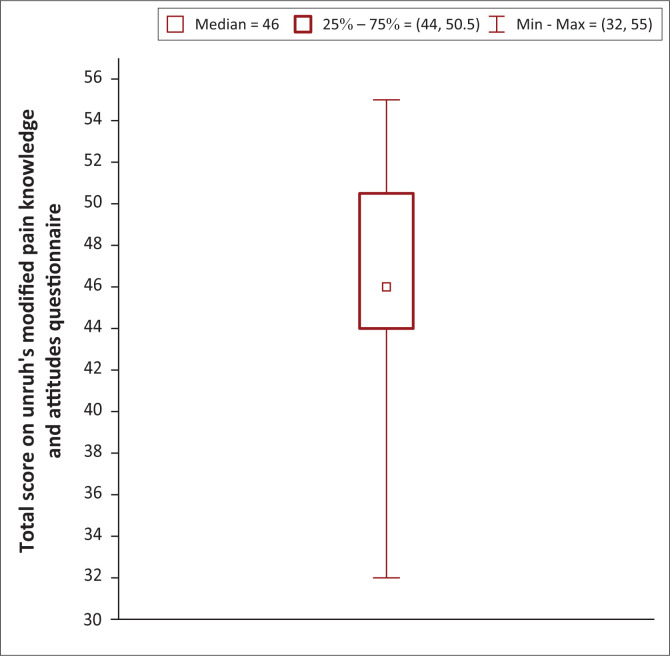
Total median scores (*n* =104).

As shown in Figure 1, the median total score on the questionnaire was 46 out of 57 (80.7%), with an IQR of 44–50.5 (77.2% – 88.6%).

**FIGURE 2:**
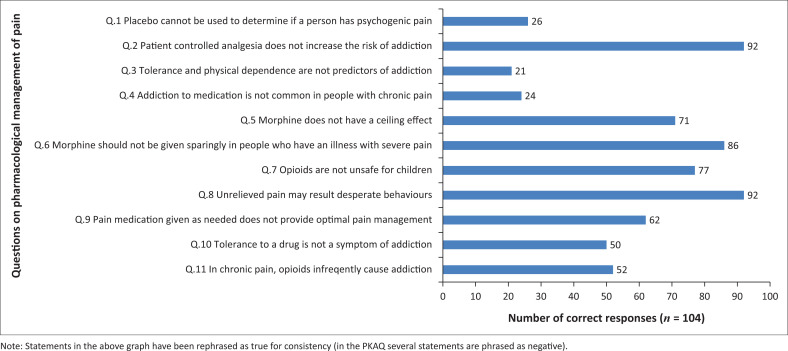
Number of participants correctly answering each question in the section on the pharmacological management of pain (*n* = 104).

1. *Physiological basis of pain*: The participants scored a median of 7 (87.5%) correct out of the 8 questions regarding the physiological basis of pain with an IQR of 6–8. Only 57 (55%) of the students correctly identified that there is NOT a predictable relationship between the extent of an injury and the person’s perception of pain (question 1). Furthermore, 37 (36%) of the participants incorrectly believed that the intensity of pain is its most important quality (question 4).
2. *Psychological factors of pain*: The participants scored a median of 11 (84.6%) correct out of the 13 questions with an IQR of 11–12. There were two questions on which participants performed poorly in this section. First, asking whether a person’s statement of pain should always be taken at face value (question 10) was incorrectly selected as untrue by 41 of the students (39%). Second, a true statement that deliberate faking of pain is rare amongst people with pain was incorrectly selected as false by 38 (36%) of the students.
3. *Developmental changes in pain*: The participants scored a median of 8 (88.9%) correct out of the 9 questions (IQR = 7–9). There was only one question in which participants performed poorly. This question asked whether a child who is playing after surgery may have pain. Twenty-four of the participants (23%) incorrectly indicated that this statement was NOT true.
4. *Assessment and measurement of pain*: The participants scored a median of 6 (75%) correct out of the 8 questions (IQR = 5–7). There were two questions on which participants performed poorly in this section. For question 35, asking participants whether blood pressure, heart rate, respiration and sweating were good measures of postoperative pain was incorrectly identified as true by 90 (87%) of the participants. For question 38, asking whether numerical pain rating scales are the gold standard for measurements in all adults and children was incorrectly indicated as true by 34 (33%) of the participants.
5. *Pharmacological management of pain*: The participants scored a median of 6 (55%) correct out of the 11 questions with an IQR of 4–8. There were several questions which were problematic in this section on pharmacological management of pain (Figure 2). The worst performance was on question 47, asking if a person who has developed tolerance and physical dependence on pain medication is likely to develop addiction – this was incorrectly answered as true by 83 (80%) of the participants. Question 46 indicating that addiction to medication is common amongst people with chronic pain was incorrectly answered as true by 80 (77%) of the participants. Finally, question 49 stating that placebos can be used to determine if a person has psychogenic pain was incorrectly answered as true by 78 (75%) of the participants.
6. *Cognitive/behavioural methods of pain relief*: The participants scored a median of 8 (100%) correct out of the 8 questions with an IQR of 7–8. The only question on which participants scored poorly was question 53 on which 22 (21%) incorrectly indicated that reinforcement of coping with pain is not an important treatment intervention.

**TABLE 1:** Results of the different sections on the questionnaire reported as medians with interquartile range.

Section on questionnaire	Results		Results
	Median	IQR	Median (%)
1. Physiological basis of pain (x/8)	7	6–8	87.5
2. Psychological factors of pain (x/13)	11	11–12	84.6
3. Developmental changes pain perception (x/9)	8	7–9	88.9
4. Assessment and measurement of pain (x/8)	6	5–7	75
5. Pharmacological management of pain (x/11)	6	4–8	54.6
6. Cognitive or behavioural methods of pain relief (x/8)	8	7–8	100
**Total Score (x/57)**	**46**	**44–50.5**	**80.7**

IQR, interquartile range.

## Discussion

Despite pain being the most common symptom that patients present with to hospital, healthcare workers inadequately assess and treat pain.^5^ This study evaluates the pain knowledge and attitudes of 2018 final-year medical students (104 students, 44.8%). Our sample size allows 90% confidence in the findings. The age and gender distribution of the participants (66% female, 24 years, IQR = 23–25 years) was similar to the age and gender distribution of the whole class (63% female, 24 years, IQR = 24–25 years).

There is no gold standard instrument currently used to assess knowledge, perceptions and attitudes to pain management. Knowledge about pain management amongst nursing and medical students is consistently generally poor, despite the diversity of standardised instruments used to evaluate it.^1^ The Unruh’s MPKAQ was used in 2007 at UCT in a study which looked at the entire final year of health science students. It was modified and piloted at the time and shown to be comparable to the original Unruh’s Pain Knowledge and Attitudes and was thus used again in our study. Instruments used in studies around the world to evaluate pain knowledge and attitudes of healthcare professionals tend to ask very similar type questions.^1^ The particular strength of this tool is its coverage of a broad range of topics, including physiological basis of pain, psychological factors of pain, developmental changes in pain perception, assessment and measurement of pain, pharmacological management of pain, and cognitive or behavioural methods of pain relief.

Because of the high prevalence of pain and its negative impact, previous authors have indicated that an appropriate pass mark on the MPKAQ should be set at 75%, a mark classified as a ‘first class’ mark at UCT.^20^ The selection of this pass mark has been motivated for based on the frequency with which healthcare professionals will encounter and need to manage pain, suggesting that expertise is required in pain management on graduation. It was encouraging to note that the participants in this survey surpassed this mark with an overall score of 80.7%. This result is similar to those of a previous study conducted in final-year health science students at UCT in 2007^3^ where the final-year medical students (*n* = 35) scored 79% on the same instrument. However, evaluation of the current study participants’ performance on the subsections of the questionnaire identifies areas of concern.

The section on the pharmacological management of pain was the most poorly answered with a median score of 55%. This score is worse than the score obtained by final-year medical students who participated in a 2007 study at UCT who scored 64% for this section.^3^ The question relating to an individual who has developed tolerance and dependence being likely to develop addiction was incorrectly answered by 80% of the participants. The question that addiction is common amongst people with chronic pain was also incorrectly answered by 77% of participants. Opioid use in non-cancer pain does not commonly result in addiction; a Cochrane review of opioids in non-cancer pain cites an incidence of addictive behaviour to be in the region of 0.27%.^25^ These misconceptions about addiction are not unusual. In the United States, medical residents were found to underuse pain scales and opioid-equivalence tables, under-prescribe patient-controlled analgesia and overestimate the risk of addiction.^26^ The need for specific education and training with regard to opioid use in the curriculum is important. Evidence-based guidelines on opioid prescribing and education on the topic has changed in the last 10 years. For example, there is limited evidence for using opioids as therapy for chronic, non-cancer pain beyond 16 weeks’ duration; however, its use in acute pain management is indicated.^26^ Potential under-treatment of acute pain with opioids has severe negative consequences for the patient, including increased morbidity, development of chronic postoperative pain, impaired function, poor recovery from surgery, poor quality of life and increased medical costs for chronic pain management.^9^ This poor performance on the pharmacological questions amongst these future prescribers highlights an important gap in the medical school curriculum. It is imperative that medical school curricula remain up-to-date with evidence-based guidelines. At the time of this study, the curriculum at UCT did not have a dedicated pain module for students. As discussed in the introduction, a fragmented approach to pain teaching may be contributing to the poor responses recorded.

On the questions relating to cognitive or behavioural methods of pain relief, the median score was 100%. However, respondents performed poorly on the question regarding the use of reinforcing coping with pain as an important treatment strategy. Understanding the biological processes that are thought to underpin pain has been shown to reduce pain itself.^27^ Developing an understanding of pain to improve the ability to cope and participate in meaningful life roles has been termed Pain Neuroscience Education.^27^ Pain Neuroscience Education aims to increase the patient’s knowledge of pain-related biology; in particular, the key message is that pain is not an accurate measure of tissue damage. Improving understanding of pain has been shown to decrease pain catastrophising and impart reduction in pain and disability.^27^

In 2016, participants in a survey of chronic pain knowledge in fifth-year medical students at the University of Stellenbosch scored poorly on an evaluation of pain knowledge with an overall score of 41%.^21^ The study used a different data collection tool to that of the present study, comprising 18 questions on basic definitions, classification and management of chronic pain.^21^ These results support those of the present study, suggesting that undergraduate South African medical students seem to lack adequate pain knowledge to manage patients presenting with chronic pain in particular.

A similar study amongst South African sports physiotherapists, where the same data collection instrument, the MPKAQ, was used, found that 85.5% of the responding physiotherapists had inadequate pain knowledge and attitudes (scored < 75%), with a mean score of 65.5%.^28^ The lowest scores obtained were for the sections on Assessment and Measurement (47.7%) and Developmental Changes in pain (58.84%).^21^ The authors noted that students who studied in their first language scored significantly higher in the physiology section than those who were studying in their second language.^28^ This highlights the importance of teaching students in their first language yielding a better understanding of the subject. At UCT, English is the medium of instruction, with the possibility that the majority of students are studying in their second language.

## Limitations

There are several potential limitations to this study. The Unruh’s MPKAQ was used. While this tool is not commonly used, it resembles many pain questionnaires with a broad coverage of topics indicated as core in the International Association for the Study of Pain curricular guidelines.^1^ While the sample size was adequate for 90% confidence, there is still a risk of recruitment bias. Students with limited knowledge of pain, or for whom English is not their first language, may have chosen not to participate. In addition, the results cannot be generalised beyond students studying at this institution and receiving the same curriculum.

## Recommendations

In line with international recommendations, the undergraduate health science students’ curriculum should include a dedicated pain module with the scaffolding of pain into related topics and clear documentation of pain teaching with defined hours allocated.^17^ Scaffolding of pain into the curriculum would include teaching pain management within each specialty that students are exposed to, i.e., pain in obstetrics, urology or orthopaedics. This would result in students learning how to integrate pain management into a patient-centred treatment approach.

## Conclusion

The assessment and pharmacological management of pain are two important areas in which the participants in this study seemed deficient. Pharmacological management of pain is a crucial tool used by doctors in the holistic management of pain. Similar to worldwide trends, South African undergraduate pain education seems to need prioritising.
